# Supplementary material for: Consistent and correctable bias in metagenomic sequencing experiments
Source: eLife. 2019 Sep 10;8:e46923. doi: 10.7554/eLife.46923 (PMC6739870; doi:10.7554/eLife.46923)
Supplement: Transparent reporting form [file elife-46923-transrepform.docx]

***eLife’s* transparent reporting form**

We encourage authors to provide detailed information *within their submission* to facilitate the interpretation and replication of experiments. Authors can upload supporting documentation to indicate the use of appropriate reporting guidelines for health-related research (see [EQUATOR Network](http://www.equator-network.org/)), life science research (see the [BioSharing Information Resource](https://biosharing.org/)), or the [ARRIVE guidelines](http://www.plosbiology.org/article/info:doi/10.1371/journal.pbio.1000412) for reporting work involving animal research. Where applicable, authors should refer to any relevant reporting standards documents in this form.

If you have any questions, please consult our Journal Policies and/or contact us: [editorial@elifesciences.org](mailto:editorial@elifesciences.org).

**Sample-size estimation**

- You should state whether an appropriate sample size was computed when the study was being designed
- You should state the statistical method of sample size computation and any required assumptions
- If no explicit power analysis was used, you should describe how you decided what sample (replicate) size (number) to use

Please outline where this information can be found within the submission (e.g., sections or figure legends), or explain why this information doesn’t apply to your submission:

We are using data from the experiments of Brooks et al 2015 and Costea el al 2017. We state the sample size and the rationale for the study design of the original studies in the Results and Methods.

**Replicates**

- You should report how often each experiment was performed
- You should include a definition of biological versus technical replication
- The data obtained should be provided and sufficient information should be provided to indicate the number of independent biological and/or technical replicates
- If you encountered any outliers, you should describe how these were handled
- Criteria for exclusion/inclusion of data should be clearly stated
- High-throughput sequence data should be uploaded before submission, with a private link for reviewers provided (these are available from both GEO and ArrayExpress)

Please outline where this information can be found within the submission (e.g., sections or figure legends), or explain why this information doesn’t apply to your submission:

Information about replicates are described in the Results and Methods (as well as in the referenced original studies Brooks et al (2015) and Costea et al (2017)). All data processing and analysis is described in the Methods and shown in a fully reproducible manner in the manuscript’s Github repositories (<https://github.com/mikemc/mgs-bias-manuscript> and <https://github.com/mikemc/metacal/releases/tag/v0.1.0-manuscript>). The reports in this repository illustrate important features of the data described in the manuscript, such as cross-sample contamination which required filtering of some data.

**Statistical reporting**

- Statistical analysis methods should be described and justified
- Raw data should be presented in figures whenever informative to do so (typically when N per group is less than 10)
- For each experiment, you should identify the statistical tests used, exact values of N, definitions of center, methods of multiple test correction, and dispersion and precision measures (e.g., mean, median, SD, SEM, confidence intervals; and, for the major substantive results, a measure of effect size (e.g., Pearson's r, Cohen's d)
- Report exact p-values wherever possible alongside the summary statistics and 95% confidence intervals. These should be reported for all key questions and not only when the p-value is less than 0.05.

Please outline where this information can be found within the submission (e.g., sections or figure legends), or explain why this information doesn’t apply to your submission:

All analyses are described in the Methods and shown explicitly and reproducibly as R functions and R-markdown documents in the manuscript’s GitHub repositories (<https://github.com/mikemc/mgs-bias-manuscript> and <https://github.com/mikemc/metacal/releases/tag/v0.1.0-manuscript>). Figures show individual (raw) data points when appropriate. P-values for the fraction of bias explained by 16S CN via permutation test are included in the Results. In all other cases, to demonstrate the validity of our inferences, we show raw data with our parameter estimates, and we report exact measures of model fit such as the number of model parameters and mean-squared error. Our method for obtaining point estimates and standard errors for the bias is described in the methods and implemented in the ‘metacal’ package.

(For large datasets, or papers with a very large number of statistical tests, you may upload a single table file with tests, Ns, etc., with reference to sections in the manuscript.)

**Group allocation**

- Indicate how samples were allocated into experimental groups (in the case of clinical studies, please specify allocation to treatment method); if randomization was used, please also state if restricted randomization was applied
- Indicate if masking was used during group allocation, data collection and/or data analysis

Please outline where this information can be found within the submission (e.g., sections or figure legends), or explain why this information doesn’t apply to your submission:

Described in the Results and Methods, and explicitly described in the analysis code and reports at the manuscript’s Github repository (<https://github.com/mikemc/mgs-bias-manuscript>).

**Additional data files (“source data”)**

- We encourage you to upload relevant additional data files, such as numerical data that are represented as a graph in a figure, or as a summary table
- Where provided, these should be in the most useful format, and they can be uploaded as “Source data” files linked to a main figure or table
- Include model definition files including the full list of parameters used
- Include code used for data analysis (e.g., R, MatLab)
- Avoid stating that data files are “available upon request”

Please indicate the figures or tables for which source data files have been provided:

The raw data for our analysis is available in the “Additional files” at http://www.biomedcentral.com/1471-2180/15/66 and in European Nucleotide Archive study accession PRJEB14847. The processed data, along with code to obtain and process the raw data and to perform all statistical analyses, are available on GitHub at <https://github.com/mikemc/mgs-bias-manuscript> and <https://github.com/mikemc/metacal/releases/tag/v0.1.0-manuscript>.
